# Age-Related Neurochemical Changes in the Vestibular Nuclei

**DOI:** 10.3389/fneur.2016.00020

**Published:** 2016-03-03

**Authors:** Paul F. Smith

**Affiliations:** ^1^Department of Pharmacology and Toxicology, School of Medical Sciences and Brain Health Research Centre, University of Otago, Dunedin, New Zealand

**Keywords:** vestibular nucleus complex, aging, monoamines, glutamate, GABA, nitric oxide

## Abstract

There is evidence that the normal aging process is associated with impaired vestibulo-ocular reflexes (VOR) and vestibulo-spinal reflexes, causing reduced visual acuity and postural instability. Nonetheless, the available evidence is not entirely consistent, especially with respect to the VOR. Some recent studies have reported that VOR gain can be intact even above 80 years of age. Similarly, although there is evidence for age-related hair cell loss and neuronal loss in Scarpa’s ganglion and the vestibular nucleus complex (VNC), it is not entirely consistent. Whatever structural and functional changes occur in the VNC as a result of aging, either to cause vestibular impairment or to compensate for it, neurochemical changes must underlie them. However, the neurochemical changes that occur in the VNC with aging are poorly understood because the available literature is very limited. This review summarizes and critically evaluates the available evidence relating to the noradrenaline, serotonin, dopamine, glutamate, GABA, glycine, and nitric oxide neurotransmitter systems in the aging VNC. It is concluded that, at present, it is difficult, if not impossible, to relate the neurochemical changes observed to the function of specific VNC neurons and whether the observed changes are the cause of a functional deficit in the VNC or an effect of it. A better understanding of the neurochemical changes that occur during aging may be important for the development of potential drug treatments for age-related vestibular disorders. However, this will require the use of more sophisticated methodology such as *in vivo* microdialysis with single neuron recording and perhaps new technologies such as optogenetics.

## Age-Associated Changes in Vestibular Function

Aging in humans has been thought to be associated with an increasing impairment of the vestibulo-ocular reflexes (VOR) and vestibulo-spinal reflexes, which results in reduced visual acuity and postural instability (1–14). The prevalence of dizziness and vertigo has been estimated at 30% in people over the age of 60, and dizziness in the elderly is associated with a high risk of falls (15, 16). Nonetheless, there is disagreement about VOR impairment, in particular. A recent study of the VORs using the video head impulse test (vHIT) for all six semi-circular canals reported that, although gain decreased with high head velocities, it was largely unaffected in healthy adults in the 80–89 years’ age group (17). Similar results have recently been reported by Matiño-Soler et al. (18), who observed that the horizontal VOR gain was stable up until 90 years of age and then decreased thereafter. McGarvie et al. (17) suggested that cerebellar compensation, in the form of VOR plasticity, may be responsible for the preservation of VOR function despite aging. On the other hand, Li et al. (19) reported that horizontal VOR gain remained stable from 26 to 79 years of age and then significantly declined (people over the age of 80 had an approximately eightfold increase in the odds of having a VOR gain <0.80). Kim and Sharpe (20) also found that the vertical VOR was relatively preserved in the elderly, although vertical smooth pursuit eye movement, eye-head tracking, and VOR cancelation during intentional head movement, were impaired. Studies of perceptual threshold levels related to the horizontal VOR have suggested that there may be little difference between young and older adults (21), although some dynamic visual acuity studies suggest otherwise (22). Otolithic function, evaluated using ocular and cervical vestibular-evoked myogenic potentials (o- and c-VEMPs), has been reported to decline with age (19, 23–26). Vestibulo-sympathetic reflexes have also been reported to be impaired with increasing age, which can lead to an increase in orthostatic hypotension (27, 28).

There is increasing evidence that age-related changes in vestibular function result in cognitive deficits (29–31). Cyran et al. (31) studied the functional connectivity of a vestibular cortical network (i.e., the superior, middle, and inferior frontal and temporal gyri, the lingual gyrus, insula, superior and inferior parietal lobe, parietal operculum, posterior cingulate gyrus, cuneus, thalamus, and cerebellar tonsil) in relation to age, using a tensor independent component analysis of fMRI data acquired in response to galvanic vestibular stimulation. They found that the functional connectivity of the network decreased with age, which they suggested was due not to structural deterioration but to functional changes; the somatosensory sensory networks, on the other hand, remained relatively intact. Recently, several large epidemiological studies have implicated vestibular dysfunction in the development of cognitive deficits in elderly humans (29, 30, 32). Although these data are based on surveys and therefore necessarily correlational in nature, they are consistent with the results of clinical studies in humans [e.g., see Ref. (33, 34) for a review] and experimental studies in animals (35–40), which have shown that vestibular dysfunction results in cognitive impairment, especially related to spatial memory.

Age-related vestibular impairment has often been attributed to a degeneration of the peripheral vestibular receptor hair cells or to changes in the number of neurons in Scarpa’s ganglion or the brainstem vestibular nucleus complex (VNC). Many studies have reported that the hair cells and their afferent connections decrease with age (41–50). Nonetheless, some studies have found no significant age-related differences in the number of hair cells in the crista ampullaris of aging gerbils (51) or the human utricle (52) and others have found no significant differences in the number of neurons in Scarpa’s ganglion (53, 54). Neuronal loss has been reported in the human VNC (55–58) and in the VNCs of some animal species [e.g., Ref. (59)]. However, Fernandez et al. (60) could find no significant age-related decrease in the number of neurons in the golden hamster. Johnson and Miquel (61) analyzed the ultrastructure of the rat lateral vestibular nucleus at 4 weeks, 6–8 weeks, 6–8 months, and 18–20 months of age, and found a number of age-related changes that increased in frequency with increasing age, including nuclear membrane invaginations, disorganized endoplasmic reticulum, rod-like nuclear inclusions, and lipofuscin-like cytoplasmic dense bodies. In addition, the oldest age group exhibited axonal degeneration and dendritic swelling. Takeuchi et al. (62) have also reported dystrophic axon terminals in the VNC of 360-day-old gerbils. The cytoplasms were found to contain neurofilaments and vesicles with membranous granular substances. Therefore, it is possible that there is age-related structural deterioration in the VNC even without neuronal loss itself.

In summary, there is increasing evidence that the human VORs are largely intact, at least until approximately 80 years of age. Ocular and cervical vestibular-evoked myogenic potentials (o- and c-VEMPs), on the other hand, appear to decline more obviously with age, and there is epidemiological evidence at least to suggest that any decline in vestibular function with age is associated with cognitive deficits. The evidence relating to structural deterioration of the peripheral hair cells, and neuronal loss in Scarpa’s ganglion and the VNC, is divided, although there may be morphological changes in the VNC irrespective of neuronal loss.

The apparent discrepancies between the results of the different functional and structural studies in aged animals and humans suggest considerable variability in the effects of aging on the vestibular system. One obvious explanation for this is species differences. Another possibility is that, even within a single species, some of this variability is the result of differences in a combination of genetic and environmental influences on the way that the vestibular system ages and the extent to which it is capable of adaptive plasticity in response to aging. In this respect, it is important to note that Radtke-Schuller et al. (63) have recently reported that the cholinergic vestibular efferent neurons, which provide feedback to the peripheral vestibular system, do not degenerate with age. By contrast, cochlear efferent neurons do degenerate with age.

It is reasonable to assume that functional changes in the vestibular nucleus that are associated with either vestibular impairment or plasticity that prevents it, would be the result of neurochemical changes and that these would constitute a neurochemical signature of the aged vestibular nucleus. The aim of this review is to summarize and critically evaluate what is currently known on this topic.

## Neurochemical Changes in the Vestibular Nucleus with Age

By contrast with the functional and neuroanatomical studies of the vestibular system, there are relatively few neurochemical studies of the VNC in relation to aging.

### Monoamines

The VNC has been shown to receive noradrenergic inputs from the locus coeruleus and the response of VNC neurons to glutamate appears to be modulated by noradrenaline (NA) via α_2_ receptors [see Ref. (64, 65) for reviews]. NA also appears to modulate the response to GABA via α_2_ receptors and β receptors (66). Likewise, the VNC receives serotonergic projections from the dorsal raphe nucleus and VNC neurons appear to have 5-hydroxytryptamine (5-HT)_1A_, 5-HT_1B_, and 5-HT_2_ receptors [see Ref. (64, 65) for reviews]. VNC neurons also respond to dopamine (DA) via “D_2_-like” receptors (i.e., D_2_, D_3_, and D_4_ receptors). There is evidence that DA depolarizes medial vestibular nucleus (MVN) neurons by acting on presynaptic “D_2_-like” receptors to inhibit the release of GABA from inhibitory interneurons [see Ref. (65) for a review].

Cransac et al. (67) studied the levels of NA, 5-HT, and DA and their metabolites in the MVN of rats at 4, 21, and 24 months of age, using homogenized micropunch samples and high performance liquid chromatography (HPLC). They found a decrease in NA with age and an increase in the ratio of its metabolite, 3-methoxy, 4-hydroxyphenylglycol (MHPG), to NA. By contrast, 5-HT and its metabolite, 5-hydroxyindoleacetic acid (5-HIAA), increased in the MVN with age while DA and 3,4-dihydroxyphenylacetic acid (DOPAC) levels remained unchanged. Di Mauro et al. (66) have suggested that a decrease in the NA content of the VNC could be responsible for deterioration of vestibular function with age.

### Amino Acids

The excitatory and inhibitory amino acids are among the most important neurotransmitters in shaping the response of VNC neurons. Every subtype of glutamate receptor is expressed in the VNC (α-amino-3-hydroxy-5-methyl-4-isoxazolepropionic acid (AMPA), *N*-methyl-d-aspartate (NMDA) and the metabotropic glutamate receptors), and glutamate is the primary neurotransmitter used in the synapses between the vestibular nerve and VNC neurons [see Ref. (64, 65) for reviews]. Similarly, many VNC neurons have γ-aminobutyric acid (GABA) receptors of both the GABA_A_ and GABA_B_ subtypes, and the GABA_A_ receptor is thought to primarily mediate commissural inhibition between the bilateral VNCs. Glycine, acting on glycine receptors, is also important in inhibitory neurotransmission in the VNC [see Ref. (64, 65) for reviews].

**Figure 1:**
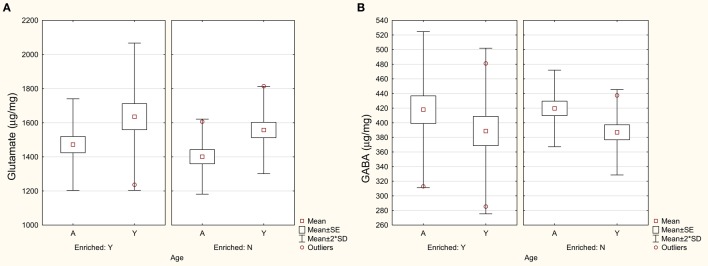
**Levels of glutamate (A) and GABA (B) in the VNC in aged (A) and young (Y) rats housed in either an enriched environment (“Y”) or not (“N”)**. Symbols represent means with the SE and SD for the mean. Modified from Liu et al. (69).

Him et al. (68) reported that the responses of MVN neurons to NMDA and AMPA were similar in brainstem slices from young (3 months of age) and aged rats (24 months of age), suggesting no change in the sensitivity of these glutamate receptor subtypes. Liu et al. (69) compared glutamate levels in the VNCs of rats at 4 and 24 months of age, using homogenized samples and HPLC, and found that glutamate levels significantly decreased with age (see Figure 1A); by contrast, there was no such decrease in the cerebellum. Since Him et al. (68) measured only the response of MVN neurons to NMDA and AMPA, and Liu et al. (69) measured only the levels of glutamate, the results of these two studies are not necessarily incompatible. For example, it is possible that AMPA and NMDA receptors upregulated or increased their sensitivity to glutamate in response to a decrease in its availability, resulting in an approximately normal response to those agonists. However, neither of these studies allows the neurochemical changes to be attributed to any specific function within the VNC. Therefore, the functional significance of these results remains unclear.

Him et al. (70) reported that neurons in the MVN from aged (24 months old) rats exhibited an increased sensitivity to the GABA_A_ receptor agonist, muscimol, which they suggested might be a compensatory change in response to a loss of neurons within the MVN. Giardino et al. (71) detected increased levels of glutamic acid decarboxylase (GAD) in the 24-month-old rat VNC, and concluded that this may reflect an increased synthesis of GABA in the aged VNC. However, Liu et al. (69), again using homogenized samples and HPLC, found no significant change in GABA levels in the VNC or cerebellum with aging in the rat (Figure 1B).

In the only study of age-related changes in glycine receptors in the VNC to date, Nakayama et al. (72) demonstrated a large decrease in strychnine binding in the VNC as a function of age (3, 18, and 26 months). The amount of strychnine binding in the 26-month-old rat was approximately 50% of that in the 3-month-old rat. Once again, the functional significance of these changes is unknown. However, Nakayama et al. (72) speculated that an increased glycine synthesis might occur in order to prevent such a large decrease in glycine receptors from causing functional impairment.

### Other Neurochemical Changes with Age

Sulaiman and Dutia (73) showed that many MVN neurons in brainstem slices are inhibited by δ-opioid receptor agonists such as [d-Ala2, d-Leu5]-enkephalin (DADLE) and [d-Pen2, Pen5]-enkephalin (DPLPE), an effect that was blocked by the δ-receptor antagonist, naltrindole. Interestingly, they found that the inhibitory effects of DADLE increased with age, although the oldest animals used were only 160–180 g in weight.

**Figure 2:**
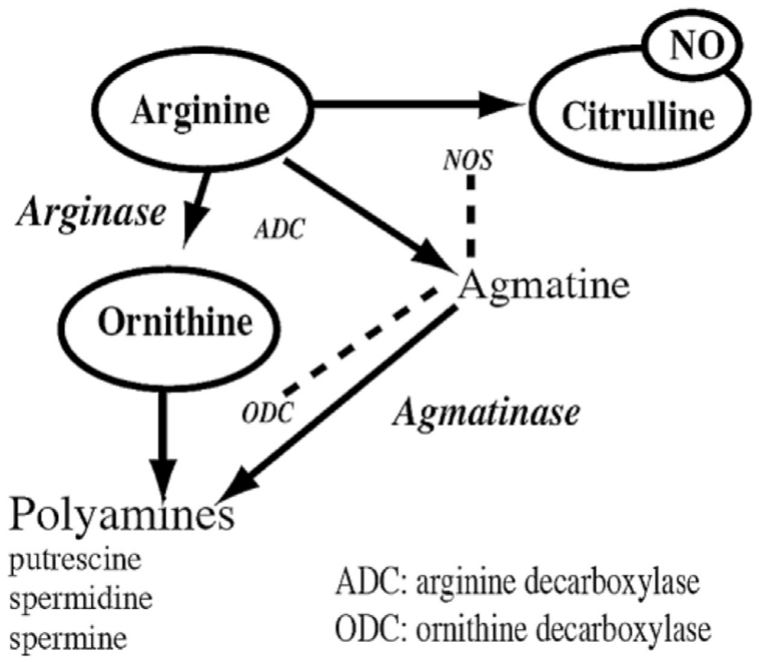
**The arginine metabolic pathway showing the conversion of l-arginine to the neurotransmitter, nitric oxide (NO), and l-citrulline, by nitric oxide synthase (NOS), of which there are three isoforms; the conversion of l-arginine to agmatine by arginine decarboxylase (ADC), which is then converted to polyamines such as putrescine, spermidine, and spermine by agmatinase and ornithine decarboxylase (ODC); and the conversion of l-arginine to l-ornithine by arginase, which is then converted to the same polyamines**. Glutamate is one of the end products of l-arginine, and glutamate serves as a precursor for the synthesis of GABA. Reproduced from Smith et al. (82) with permission from Elsevier.

Liu et al. (69) were interested in analyzing the l-arginine metabolic system in the VNC and cerebellum of aged (24 months old) and young (4 months old) rats. Some of the rats were housed in a standard environment, and others were housed in an enriched environment, with running wheels and toys. They employed homogenized samples, HPLC and liquid chromatography/mass spectrometry (LC/MS/MS) to quantify the concentrations of l-arginine, l-citrulline, l-ornithine, agmatine, putrescine, spermidine, spermine, as well as glutamate and GABA (the latter as previously mentioned). These neurochemicals are all related and part of the l-arginine metabolic pathway (see Figure 2). l-arginine is a semi-essential amino acid metabolized by nitric oxide synthase (NOS) in order to produce nitric oxide (NO) and l-citrulline (74). NO is non-conventional neurotransmitter that is important for synaptic plasticity and learning and memory; however, it is also a free radical, and therefore in excessive amounts it can be neurotoxic [see Ref. (75) for a review]. There is a great deal of evidence that NO is implicated in both the normal aging process and age-related neurodegenerative processes [(76, 77); see Ref. (78, 79) for reviews]. The polyamines putrescine, spermidine, and spermine are down-stream metabolites of l-arginine (see Figure 2).

**Figure 3:**
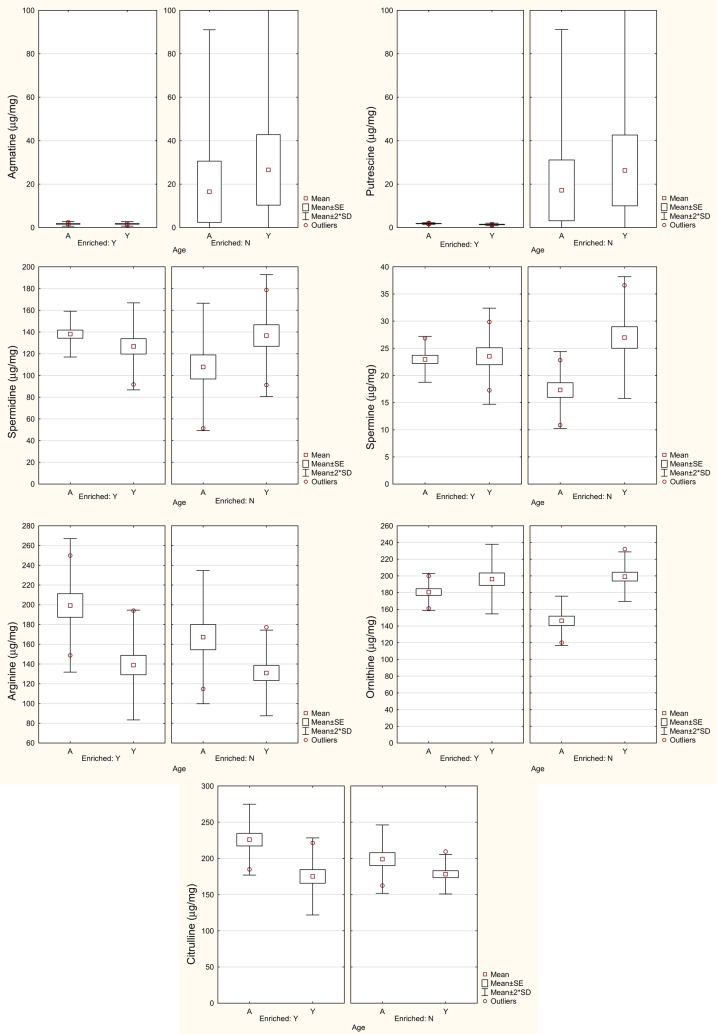
**Levels of agmatine, putrescine, spermidine, spermine, l-arginine, l-ornithine, and l-citrulline in the VNC in in aged (A) and young (Y) rats housed in either an enriched environment (Y) or not (N)**. Symbols represent means with the SE and SD for the mean. Modified from Liu et al. (69).

Liu et al. (69) found that in the VNC, putrescine, l-arginine, and l-citrulline increased significantly with age, while spermine and l-ornithine decreased (see Figure 3). In the cerebellum, spermidine and l-citrulline increased significantly with age, while spermine decreased. Linear discriminant analysis (LDA) was used to show that age could be predicted from a subset of these neurochemicals. For the VNC, the LDA could predict age with 100% accuracy from the levels of putrescine, spermidine, spermine, l-citrulline, glutamate, and GABA. For the cerebellum, age could be predicted with 93% accuracy from the levels of spermine and spermidine only.

l-citrulline (the coproduct of NO) was significantly higher in the aged VNC and cerebellum, which is consistent with the increase in NO in the aged cerebellum that has been reported previously (80). Mistry et al. (81) reported that l-arginine concentrations in the cerebellum were not significantly different between young (3–5 months old) and aged (18–22 months old) male rats, which is consistent with the results of Liu et al. (69).

**Figure 4:**
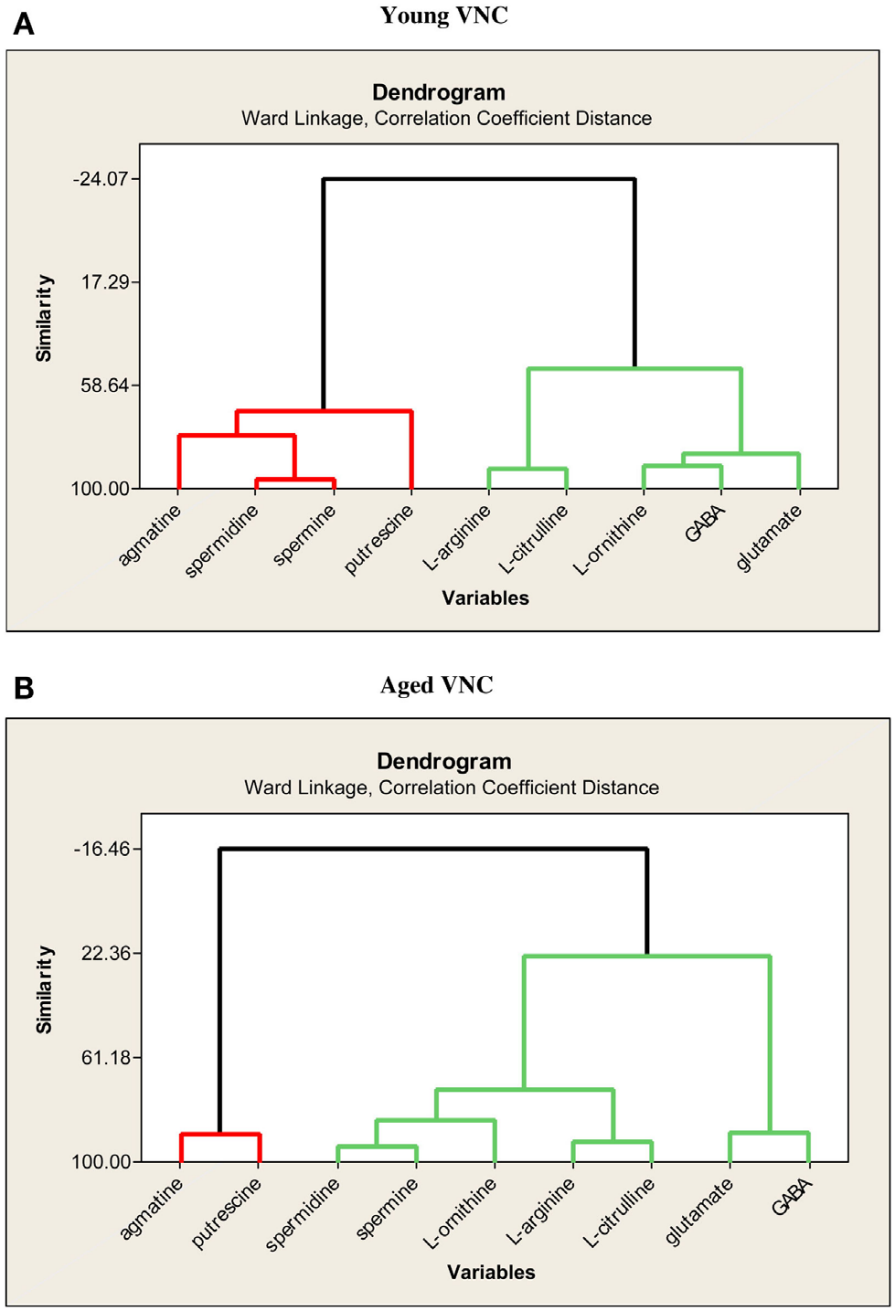
**Dendrograms showing the similarities in the degree of expression of the nine neurochemical variables in the VNC of young (A) and aged (B) rats**. Reproduced from Liu et al. (69) with permission from Elsevier.

In further analyses of the same data set, Smith et al. (82) used multiple linear regression in order to determine whether each variable could be predicted from the others. Age was a significant predictor variable for putrescine (*R*^2^ = 0.68), spermidine (*R*^2^ = 0.93), agmatine (*R*^2^ = 0.76), and l-ornithine (*R*^2^ = 0.50). Using cluster analyses, there were no large differences in the covariation of the different neurochemical variables between the young and aged animals, and glutamate and GABA covaried closely in both groups (see Figure 4).

**Figure 5:**
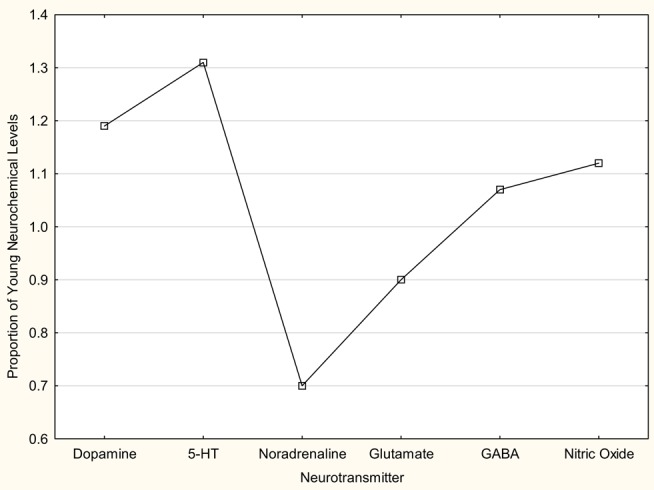
**Schematic diagram of changes in levels of dopamine, 5-HT, noradrenaline, glutamate, GABA and nitric oxide, with age**. The data regarding dopamine and 5-HT are from Table 1 in Cransac et al. (67). The data regarding noradrenaline are estimated from Figure 1 in the same paper. The data regarding glutamate and GABA are from Liu et al. (69). The nitric oxide levels are estimated from the levels of the coproduct l-citrulline (i.e., both are produced from the action of nitric oxide synthase on l-arginine), also from Liu et al. (69). In all cases, the young data are from rats at 3–4 months of age and the aged data from animals at 24 months of age. The data from Cransac et al. (67) are from the MVN whereas the data from Liu et al. (69) are from the VNC as a whole. In all cases, the aged data are expressed as a proportion of the mean values for the young data in each study, in order to make comparisons across studies possible. The changes in 5-HT, noradrenaline, glutamate, and nitric oxide were statistically significant.

In summary, there is evidence that, with aging, the levels of NA and glutamate decrease in the VNC, while those of 5-HT and NO increase (Figure 5). On the other hand, there is evidence that GABA and DA levels do not change significantly (Figure 5). The data relating to neuronal responsiveness are more difficult to interpret, since they may reflect receptor number, affinity or efficacy; however, the available data suggest that the response of VNC neurons to NMDA and AMPA receptor agonists does not change significantly, while GABA_A_ receptor and δ-opioid receptor agonists have an increased effect. There is a significant downregulation of glycine receptors in the VNC with age.

## Conclusion and Future Directions

Although vertigo and dizziness are common complaints among the elderly (15, 16), there is some disagreement as to how much of this is due to VOR dysfunction. Some recent studies have suggested that the VOR remains relatively preserved even in people over the age of 80 [e.g., Ref. (17)]. There is, on the other hand, evidence for dysfunction of o- and c-VEMPs with advancing age [e.g., Ref. (19, 23–26)]. The evidence for age-related hair cell loss and neuronal loss in Scarpa’s ganglion and the VNC is divided; however, it may be that structural deterioration occurs even without neuronal loss. One possibility is that some of the changes that lead to increased vertigo and dizziness in the elderly are related not to peripheral degeneration or even degeneration in the VNC, but to deterioration in the vestibulo-limbic and vestibulo-cortical pathways. Recent studies have shown age-related changes in the vestibulo-cortical networks and epidemiological studies have increasingly linked vestibular impairment to cognitive deficits in the elderly (29–31).

Assuming that there are structural and functional changes in VNC neurons with age that contribute to vestibular impairment, it is almost certain that these will be dictated by neurochemical changes, either causing deterioration of function or perhaps caused by that deterioration; some of these changes may even be compensatory and may help to preserve vestibular function, up to a point. Unfortunately, at this time, there are relatively few quantitative studies that can shed light on this topic. Of the studies that have been published, there is evidence for a decrease in NA and an increase in 5-HT in the VNC, with no change in DA levels (67); a decrease in glutamate levels (69) with no change in the sensitivity of AMPA and NMDA receptors (68) (Figure 5). The evidence relating to GABA is contradictory, with Giardino et al. (71) reporting an increase in GAD, possibly reflecting an increased synthesis of GABA, while Liu et al. (69) found no significant change in GABA levels (Figure 5). On the other hand, Him et al. (70) reported an increased sensitivity of MVN neurons to a GABA_A_ receptor agonist. There is evidence for a decrease in glycine receptors (72) and an increase in the sensitivity of MVN neurons to the inhibitory effects of δ opioid receptor agonists (73). Finally, Liu et al. (69) have reported complex changes in various neurochemicals that are part of the l-arginine metabolic pathway, some of which are implicated in the production of NO. Unfortunately, to completely understand the changes that are occurring in any particular neurotransmitter system, it is necessary to have information not only about neurotransmitter levels, preferably their release (see below), but also the number, affinity and efficacy of their receptors (i.e., the degree to which activation of the receptor by an agonist results in a change in neuronal function, for example, via influx of ions through linked ion channels or G protein mobilization). For example, it is possible to have no change in neurotransmitter release, no change in receptor number or affinity, however a change in efficacy. While neurotransmitter release can be measured using microdialysis *in vivo*, and receptor number can be measured using receptor binding, western blotting or immunohistochemistical methods, affinity, with spatial information, is best measured using receptor autoradiography, and efficacy requires the use of patch clamping (for ion channel-linked receptors) or GTPase assays (to measure G protein mobilization for G protein-coupled receptors). The combination of all these methods is rarely used and therefore it can be difficult to interpret the functional meaning of a decrease or an increase in a neurotransmitter without knowing the number, affinity and efficacy of the receptors.

Aside from the many differences in species and methods, e.g., different types of HPLC to analyze neurochemical levels and electrophysiological versus binding techniques to analyze receptors, most of the available studies suffer from the limitation that it is difficult, if not impossible, to relate any neurochemical changes observed to the function of specific VNC neurons (other than attributing the changes to a specific subnucleus of the VNC such as the MVN, if only that subnucleus was dissected). Furthermore, it is impossible to determine whether any observed changes are the cause of a functional deficit in the VNC as opposed to an effect of it. In fact, there are few electrophysiological studies of VNC neuronal function in aged animals. One reason for this is that aged animals are expensive and difficult to maintain for the required length of time. Twenty-two months for a rat is equivalent to approximately 65 years for a human, and therefore some animals will not survive the desired length of time. A perennial limitation of HPLC studies is that they analyze the total concentrations of neurochemicals in brain tissue, not only the components related to their role as neurotransmitters, and therefore some of the changes may reflect energy metabolism and any apparent lack of change may be due to adjustments in the neurotransmitter/non-neurotransmitter components (69). Therefore, one important future direction will be to combine microdialysis, which can measure neurotransmitter release within the VNC, with electrophysiological recording from single neurons. This is a much more difficult method than using brain homogenate samples, but will yield more specific information. Another important new direction will be to exploit optogenetic techniques in order to selectively modulate neuronal subtypes via light-sensitive proteins that have been genetically inserted into the target neurons (83), while at the same time using microdialysis to measure neurotransmitter release and electrophysiology to record functional changes.

## Author Contributions

The author confirms being the sole contributor of this work and approved it for publication.

## Conflict of Interest Statement

The author declares that the research was conducted in the absence of any commercial or financial relationships that could be construed as a potential conflict of interest.

## Abbreviations

5-HT, 5-hydroxy-tryptamine; AMPA, α-amino-3-hydroxyl-5-methyl-4-isoxazole-propionate; GABA, γ-aminobutyric acid; HPLC, high performance liquid chromatography; NMDA, *N*-methyl-d-aspartate; NO, nitric oxide; VNC, vestibular nucleus complex; VOR, vestibulo-ocular reflex.
